# REFLECTIONS FROM HOMELAB: DEVELOPING TASKS TO MEASURE UPPER-BODY FUNCTION IN A SIMULATED HOME

**DOI:** 10.1093/geroni/igz038.3413

**Published:** 2019-11-08

**Authors:** Alicia G Carmichael, Maren Wisniewski, Karen Nielsen, Natalie M Leonard, Marianthie Wank, Vineet Raichur, Richard Gonzalez

**Affiliations:** 1 University of Michigan, Ann Arbor, Michigan, United States; 2 Georgia State University, Atlanta, Georgia, United States

## Abstract

Homelike research spaces provide a unique context for studying older adulthood by blurring the boundaries between the participant’s own home and traditional laboratories. Such intermediary spaces hold promise for developing measures of functional performance with increased ecological validity. While “home labs” are becoming more accessible at institutions around the world, the research design process in these spaces contains hidden challenges that can be a barrier to entry for the uninitiated. We identify and reflect on these challenges through the lens of a recent protocol built to assess upper-body performance among older adults during activities of daily living. The U-M HomeLab served as a proving ground for four example tasks: opening a water bottle, sorting pills, tying an apron, and hanging laundry. The evolution of each task is traced through ideation, testing, and refinement, culminating in a pilot among nine community-dwelling volunteers aged 61 to 72 with upper-body pain. Based on this experience, we recommend that designers of naturalistic tasks in homelike environments give special consideration to (1) feasibility, (2) scorability, and (3) safety while carefully balancing standardization against verisimilitude. In turn, each of these elements must be grounded not only in the context of the facility itself but also in the population using that facility. Among older adults with upper body pain, considerations included remaining cognizant of fall risk, anticipating and capturing compensatory behaviors, tailoring task difficulty for a wide range of physical ability, and accounting for the impact of historically gendered divisions of labor on task performance.

